# Retinoic acid improve germ cell differentiation from human embryonic stem cells

**Published:** 2013-11

**Authors:** Liu Xuemei, Yue Jing, Xu Bei, Hu Juan, Ren Xinling, Liu Qun, Zhu Guijin

**Affiliations:** 1 *Reproductive Medicine Center, Yantai Yuhuangding Hospital, Yantai 264000, China.*; 2 *Reproductive Medicine Center, Tongji Hospital, Tongji Medical College, Huazhong University of Science and Technology, Wuhan 430030, China.*

**Keywords:** *Cell Differentiation*, *Embryonic Stem Cells*, *Germ cells*, *Retinoic acid*

## Abstract

**Background:** Creation of artificial gametes may provide a universal solution for these patients with no gametes. Stem cell technology may provide a way to obtain fully functional gametes. Retinoic acid (RA) can initiate meiosis. Several studies have demonstrated that RA can promote sperm cells differentiation from mouse embryonic stem cells (mESCs) and other cells from human embryonic stem cells (hESCs).

**Objective: **We sought to determine whether RA could promote differentiation of germ cells from hESCs.

**Materials and Methods:** hESCs were differentiated as embryoid bodies (EBs) in suspension with all-trans RA (atRA) or without atRA for 0, 1, 3, 5 and 7 days, and then the expression of VASA, SCP3, GDF9 and TEKT1 were compared by real-time PCR. The statistical differences were evaluated by one way ANOVA.

**Results: **The expression of germ cell-specific markers including the gonocyte marker VASA, the meiotic marker SCP3, and post meiotic markers, GDF9 and TEKT1, all increased in the presence and absence of RA as EB differentiation progressed. In addition, the expression of these markers increased an average of 9.3, 6.9, 7.2 and 11.8 fold respectively in the presence of RA, compared to the absence of RA, over 5 days differentiation.

**Conclusion:** Our results indicate that hESCs may have the potential to differentiate to primordial germ cells (PGCs) and early gametes. RA can improve germ cells differentiation from hESCs.

## Introduction

In present time, some infertile couples have no gametes or gonads. Additionally, age related (in women) poor quality oocyte production poses an everyday hurdle for couples wishing to conceive (1). This later group of females with advanced reproductive age represents the majority of failed in-vitro fertilization (IVF) cases, who correspond to a significant social problem (1, 2). Oocyte donation is the only option currently for this massive group of patients but does not represent a satisfying solution for different reasons. Hence, many investigators are looking for alternative treatment options such as creation of artificial gametes. Moreover, if oocytes can be efficiently produced in vitro, this will play an important role on progression in nuclear transfer and nuclear reprogramming technology (3). 

In recent years, embryonic stem cell differentiation technology has provided a new alternative (4, 5). Embryonic stem cells are able to grow and self-renew unlimitedly and have an ability to differentiate into all types of cells in the body including germ cells. In the last few years, several groups have demonstrated that mouse embryonic stem cells (mESCs) can develop into primordial germ cells (PGCs) in vitro (4-7), ever some may form early spermatids or oogonia after further culture (4, 5, 8, 9). When the haploid spermatids generated from mESCs were isolated and injected intracytoplasmically into oocytes, the diploid chromosome complement was restored (5). 

Toyooka *et al* showed that Mvh-LacZ murine germ cells (transgene with germ cell- specific mouse VASA homolog and LacZ reporter) generated in vitro from mESCs could also develop to some extent in vivo (10). Nayernia *et al* recently produced viable transgenic offspring from sperm produced from mESCs using a novel two-stage culture system (11, 12). In addition, Qing *et al* demonstrate a novel, two-step method for inducing mESCs to differentiate into oocyte-like cells using mouse ovarian granulosa cells (13). 

Investigations with human embryonic stem cells (hESCs) are more preliminary. They also show that hESCs spontaneous or induced differentiation in culture can generate PGCs and gamete formation (14-19). However, spontaneous differentiation of ESCs into germ cells is generally low and inefficient. Retinoic acid (RA), a vitamin A derivative, exerts a wide range of biological effects. It is involved in the control of cellular differentiation and cellular proliferation. RA markedly increases the number of migratory phase PGCs and slows the depletion of PGCs in vitro (20, 21). 

Furthermore, It can promote PGCs proliferation and/or survival in vitro. Recent data show that RA signaling determines germ cells fate in mice (22-24). It is responsible for the induction of germ cell meiosis in the developing ovary, and in the fetal testis, this process is inhibited by a RA metabolizing enzyme. In addition, RA has an important role in the process of mESCs differentiated into germ cells (5, 11, 25). To develop methods to efficiently differentiate germ cells from hESCs, we tested whether RA might also improve the differentiation of germ cells from hESCs. Some data show that RA can enhance the differentiation of hESCs to somatic cells, such as smooth muscle cells, motor neurons and insulin-producing cells (26-28). 

However, the expression of germ cell-specific genes was not assayed in these studies. Here, we differentiated hESCs in the presence of RA and assayed differentiation of germ cells by analysis of gene expression. 

## Materials and methods

**hESCs culture and differentiation**

The hESC lines used in this study were the cell lines established in the Reproductive Center of Tongji Hospital named hES-8 (46, XX karyotype) and hES-18 (46, XY karyotype) with the ability to differentiate into all types of cells in the body (29) in 2005 year. With written informed consent by Tongji Hospital Research and Ethics Committee, the hESC lines were used in the research. This is a prospective study about hESCs. The undifferentiated hESCs were cultured on the mitomycin-treated mouse embryonic fibroblast (MEF) feeder layer in 5% CO_2_ in standard medium consisted of knockout DMEM (Gibco, USA), supplemented with 20% Knockout Serum Replacement (Gibco, USA), 2 mM L-glutamine (Gibco, USA), 1% non-essential amino acids (Hyclone, USA), 0.1 mM β-mercaptoethanol (Sigma, USA), 100 IU/ml penicillin (Hyclone, USA), 100 μg/ml streptomycin (Hyclone, USA) and 4 ng/ml recombinant human basic fibroblast growth factor (bFGF; Pepro Tech, Inc., USA). Colonies of hESCs were routinely passaged by mechanical disaggregation into clumps, which were replaced onto fresh mitomycin-treated MEF in fresh medium every 6 days.

Approximately 5×10^4^ (one 35mm culture dish) of undifferentiated hESCs were dissociated into clumps by mechanical methods, and then were collected and distributing into individual wells of an ultralow-attachment plate with differentiation medium consisting of knockout DMEM, supplemented with 20% fetal bovine serum, 2 mM L-glutamine, 1% non-essential amino acids, 0.1 mM β-mercaptoethanol, 100 IU/ml penicillin and 100 μg/ml streptomycin, with the exception that some wells also contained atRA (Sigma, USA). atRA is soluble in absolute ethanol and diluted to 10^-2^ M. Subsequent dilutions were made in medium with a final ethanol concentration of 0.1% (v/v) which did not affect the described system. hESCs were then differentiated as EBs in suspension for 0, 1, 3, 5 and 7 days. Subsequently, each well of EBs was collected by centrifugation at 1,000 g for 5 min and stored at -80^o^C until further analysis.

**Gene expression analysis**

**RNA extraction**

Total RNA from each sample was extracted by using the TRI reagent (Molecular Research Center Cincinnati, Inc., USA) according to the manufacturer’s instructions. 

**cDNA production (reverse transcription)**

One microgram of total RNA was reverse-transcribed to complementary DNA in a 30 μl reaction mixture containing 6μl 5 × RT buffer, 1μl 10mM dNTP, 1μl 0.5μg/μl oligo (dT)15, 0.5μl 50IU/μl ribonuclease inhibitor, and 1μl 200IU/μl M-MLV reverse transcriptase (Promega Corp., USA). The complementary DNAs were further amplified by PCR using selected primers (Table    I ).

**Quantitative PCR**

Real-time PCR was performed with an Mx3000P thermo cycler (Strata gene, CA, USA) using SYBR GREEN 1 fluorescence detection of amplified products (14, 16, 30). β-actin was used in parallel for each run as internal control. A 25 μl PCR reaction was used and included 10 mM Tris-HCI (pH 8.3), 50 mM KCI, 2 mM MgCI_2_, 200 μM dNTPs, 1.5 IU TaKaRa Taq^TM^ DNA polymerase, 0.4×SYBR GREEN 1 (Invitrogen, Basel, Switzerland) and 0.15 μM of each primer. A four-step experimental run protocol (31) was used and the amplification conditions were as follow: 95^o^C for 10 min (initial denaturation), 35 cycles of 25 s at 95^o^C (denaturation), 30 s at annealing temperature, 30s at 72^o^C (elongation), 8s at fluorescence measurement temperature (Table I). 

A melting curve was generated at the end of every run to ensure product uniformity. PCR products were run on a 1.5% agarose gel and further verified by nucleotide sequencing. For each sample, a replicate was run omitting the reverse transcription step and a template negative control was run for each primer combination. Standard curves were constructed with serial dilutuons of complementary DNA from the samples (31, 32). 

**Calculation**

After 1 day differentiation with addition of difference concentration RA (1μM, 3μM, 5μM, 7μM and 10μM RA), we evaluated the expression of VASA and SCP3 by real-time PCR to discover the optimum effect concentration of RA. The fold change in expression of each gene was calculated relative to normalized expression of hESCs with addition of 1μM RA. In the process of differentiation (for 0, 1, 3, 5 and 7 days), we compared the expression of germ cell markers in the presence and absence of optimum concentration of RA respectively by real-time PCR, to determine whether RA could improve differentiation of germ cells from hESCs. 

The fold change in expression of each gene was calculated relative to normalized expression of hESCs without addition of RA. Normalized expression values were calculated using β-actin as a reference. 

**Statistical analysis**

The data are presented as the mean± SD and statistical differences were evaluated by one way ANOVA using SPSS (13.0). P<0.05 was considered significant.

## Results

**Meiotic and post-meiotic germ cell differentiation of hESCs in vitro**

The hESCs were allowed to differentiate spontaneously to EBs in non-adherent culture for 0, 1, 3, 5 and 7 days. In the process of differentiation, we found that OCT4 expression decreased predominantly in pluripotent cell types, whereas there was a sharp increase in expression of the later germ cell lineage markers including the gonocyte marker VASA, the meiotic marker SCP3, and post meiotic markers, GDF9 and TEKT1 as EB differentiation progressed (33, 34). We observed only very low, basal levels of VASA and no expression of SCP3, GDP9 and TEKT1 in undifferentiated hESCs (0 day). 

**Figure 1:**
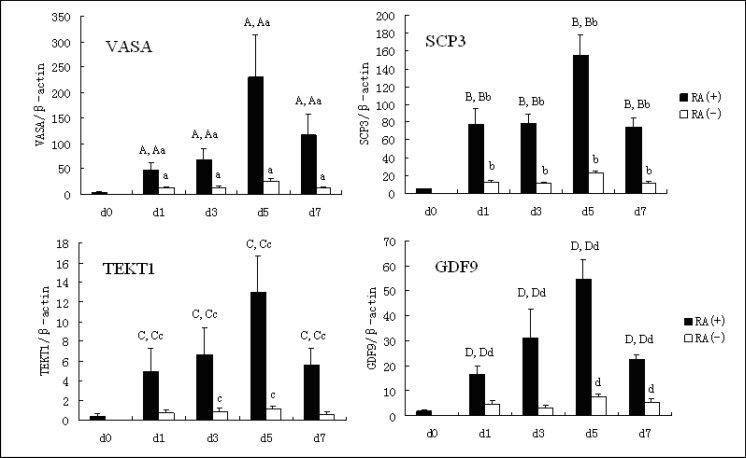
Temporal expression of markers of germ cell in EBs in the presence and absence of RA.

After 5 days of differentiation, the expression of them all increased significantly. In addition, we observed that human germ cells differentiated in vitro, expressed both the male and female genetic programs regardless of whether they were from karyotypically XX or XY hESC line. Expression of both GDF9 (an oocyte specific gene) and TEKT1 (a spermatid specific gene) was noted with differentiation of the hESC line (46 XX and 46 XY) (Figure 1). These results were similar to observations by other researchers (14, 18). Thus, we were confident that the results are qualitatively and quantitatively reproducible, even in different laboratories, using different hESC lines.

**RA induced expression of germ cell markers**

**Figure 2:**
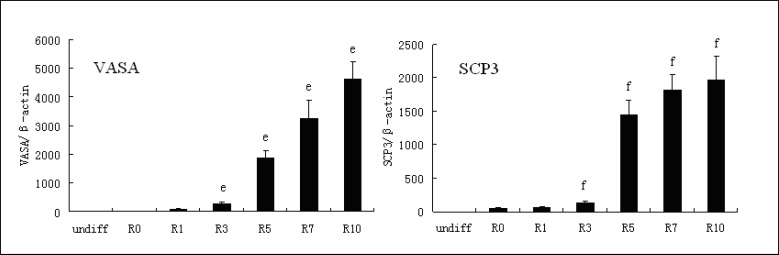
The expression of VASA and SCP3 with addition of RA.

We added RA to differentiating EBs and assayed the expression of VASA, a germ cell-specific marker that is expressed initially in gonocytes, and SCP3, a germ cell-specific marker that is expressed initially at meiosis for the first time (33, 34). We demonstrated that there is little or no expression of these markers in undifferentiated hESCs. After 1 day, we noted that spontaneously differentiated EBs showed the same levels of the markers as undifferentiated hESCs. In contrast, EBs treated with RA demonstrated a dramatic increase in expression of the germ cell markers. The expression of VASA and SCP3 increased 57 and 29 fold respectively in the presence of 10 μM RA, compared with 1 μM RA (Figure 2).

**Temporal expression of germ cell markers with RA treatment**

We examined gene expression of spontaneous differentiated EBs for 0, 1, 3, 5 and 7 days culture, in the presence and absence of 10 μM RA. Previously, RA was shown to increase differentiation of other cell lineages from hESCs. However, germ cell differentiation in vitro was not reported. Hence, we examined expression of germ cell markers as well as a marker of undifferentiated hESCs, OCT4, in the presence and absence of RA. We found that the expression of OCT4 decreased in the presence and absence of RA. 

Examination of the germ cell marker VASA, SCP3, GDF9 and TEKT1 in these experiment indicated that expression of these markers all increased in the presence and absence of RA as EB differentiation progressed. In addition, the expression of these markers increased an average of 9.3 (VASA), 6.9 (SCP3), 7.2 (GDF9) and 11.8 (TEKT1) fold respectively in the presence of RA, compared with the absence of RA, over 5 days differentiation (Figure 1).

**Table I:** Primers used for real time-PCR

**Gene**	**Primer sequences**	**Fragment size** ** (bp)**	**Annealing temperature (** ^o^ **C)**	**Fluorescence measurement temperature (** ^o^ **C)**
VASA	5’-TCTGCGAAACATAGGGGATGA-3’	315	60	85
	5’-CTGCCAGTATTCCCACAACGA-3’			
SCP3	5’-TGCAGAAAGCTGAGGAACAAG-3’	249	62	86
	5’-CTTGCTGCTGAGTTTCCATCA-3’			
GDF9	5’-CGCAGAGGTCAGGAAACTGTC-3’	315	60	87
	5’-GGCAGGTACACATGACGGTCT-3’			
TEKT1	5’-AGGCCATCCTTGACCAAGAAG-3’	236	62	89
	5’-TTTGACCTGGATCTCCTCCTG-3’			
OCT-4	5’-ACATCAAAGCTCTGCAGAAAGAACT-3’	127	60	86
	5’-CTGAATACCTTCCCAAATAGAACCC-3’			
β-actin	5’-GAGCTACGAGCTGCCTGACG-3’	416	60	91
	5’-CCTAGAAGCATTTGCGGTGG-3’			

In the process of differentiation, we found that there was a sharp increase in expression of the later germ cell lineage markers including VASA, SCP3, GDF9 and TEKT1 as EB differentiation progressed. In addition, the expression of these markers increased an average of 9.3 (VASA), 6.9 (SCP3), 7.2 (GDF9) and 11.8 (TEKT1) fold respectively in the presence of RA, compared with the absence of RA, over 5 days differentiation.

d0: undifferentiated hESCs; d1: differentiated hESCs for 1 day; d3: differentiated hESCs for 3 days; d5: differentiated hESCs for 5 days; d7: differentiated hESCs for 7 days; RA(+): the presence of 10 μM; RA(-): the absence of RA.

Statistically significant differences (p<0.05) are indicated by different letters (A, a, Aa, B, b, Bb, C, c, Cc, D, d, Dd).

A, B, C, D: the group RA (+) compared to d0;

a, b, c, d: the group RA(-) compared to d0;

Aa, Bb, Cc, Dd: the group RA(+) compared to RA(-).

The results demonstrated that there is little or no expression of VASA and SCP3 in undifferentiated hESCs and differentiated EBs for 1 day. In contrast, EBs treated with RA demonstrated a dramatic increase in expression of the germ cell markers. The expression of VASA and SCP3 increased 57 and 29 fold respectively in the presence of 10 μM RA, compared with 1 μM RA.

undiff: undifferentiated hESCs; R0: the absence of RA; R1: 1μM RA; R3: 3μM RA; R5: 5μM RA; R7: 7μM RA; R10: 10μM RA.

Statistically significant differences (p < 0.05) are indicated by different letters (e, f).

## Discussion

Formation of mature germ cells from ESCs has been relatively successful using mESCs culture (4, 5, 8, 9, 25). In the majority of these studies, identification of maturing germ cells was made possible using either integrated germ cell reporter constructs and/or cell surface markers. Recently, one study (11, 12) used a novel culture system produced viable transgenic offspring from sperm. mESCs were transfected with both reporter genes Stra8-EGFP (a RA-responsive gene expressed in pre-meiotic mouse germ cells and fused with enhanced green fluorescent protein gene construct) and Prm1-dsred (protamine1 gene fused to red fluorescent protein gene construct). 

After induction by RA, mESCs produced spermatogonial stem cells (SSCs). These cells are able to undergo meiosis, generate haploid male gametes, and may fertilize after intracytoplasmic injection into mouse oocytes. Two-cell embryos were transferred into oviducta, and live mice were born. Another study demonstrated a novel method to differentiate into oocyte-like cells. PGCs were differentiated to EB cells, and then the cells were co-cultured with ovarian granulosa cells (13). After 10 days, germ cell colonies were generated from these cells and expressed the mouse vasa homolog (Mvh) and synaptonemal complex protein (SCP) 3. These cells also expressed the oocyte-specific genes Figalpha, growth and differentiation factor (GDF)-9, and zona pellucida (ZP) 1-3 but not any testis-specific genes.

The investigations with hESCs are more preliminary. In this study, we use a panel of germ cell-specific markers to assess the ability of undifferentiated hESCs to form germ cells in vitro. Several lines of evidence suggest that this a valid approach to assay germ cell development. In particular, this approach has been used successfully to diagnose both female and male germ cell development from mESCs (4, 10). Indeed, male germ cells were sorted from differentiated ES cells expressed VASA, and when these VASA-positive cells were transplanted into the testis of mice, the mature sperm could be produced. 

In contrast, transplantation of unsorted ES cells into the testis of recipient mice resulted in teratoma formation (10). In addition, no cell types, other than germ cells, are known to express proteins such as VASA, SCP3, GDF9 and TEKT1 in evolutionarily divergent organisms. We observed that with hESCs differentiation into EBs, expression of the early gonocyte specific marker VASA was initiated together with expression of SCP3, GDF9 and TEKT1. Taken together, these results suggested that gonocyte-like germ cells that expressed VASA and other germ cell specific genes are specified during hESCs differentiation in vitro.

In this study, we observed VASA and SCP3 expression in EBs from hESCs, and the expression increased as EBs differentiation progressed. This suggests that hESCs have the ability to produce germ cells by spontaneous differentiation. In addition, we observed that human germ cells differentiated in vitro expressed both the male and female genetic programs regardless of whether they were from karyotypically XX or XY hESC line. The expression of both GDF9 (an oocyte specific gene) and TEKT1 (a spermatid specific gene) was noted with differentiation of the hESC lines (46 XX and 46 XY). During mESCs culture, markers of female germ cells are also expressed in both XX and XY cell lines (5, 10). 

The conventional hypothesis has been that germ cells are cell-autonomous and intrinsically programmed to undergo meiosis (35). However, recent studies indicate that germ cells respond to the external signal of RA and its metabolism (22, 23). Thus, in the embryonic mouse ovary, RA induces germ cells to express the pre-meiotic marker Stra-8 (stimulated by retinoic acid 8) and initiate meiosis. By contrast, in the embryonic mouse testis, RA is metabolized and inactivated by the P450 enzyme CYP26 (B1), thereby preventing early germ cell entry into meiosis and induction into the alternative pathway of mitotic arrest as G0/G1 prospermatogonia. The implication from these findings is that RA or possibly other factors affecting meiosis and gamete determination are present in the culture conditions possibly generated by male and female hESCs.

RA is a crucial signaling molecule during vertebrate development and plays key roles in cell differentiation, proliferation and apoptosis (36). It has been reported that RA induces ESCs differentiation in a dose and time-dependent manner. In mESCs, high concentration of RA (10^-5^-10^-6^ M) induces the formation of germ cells (5, 11, 25) and neural cells, while low concentration (10^-8^-10^-9^ M) promotes differentiation of smooth muscle and myocardial cells (37-39). 

In hESCs, high concentration of RA (10^-5^-10^-6^ M) induces the formation of smooth muscle cells (26) and insulin-producing cells (28). In present study, RA also regulated gene expression in a time and concentration dependent manner. Optimal level of gene expression was induced when hESCs were treated with 10 μM RA. Likewise, RA treatment produced a time dependent change in gene expression. The expression of all markers increased significantly in 5 days, and then decreased. Collectively, these data suggest that RA makes an important effect on ESCs differentiation inth germ cell lineage.

## Conclusion

Our results demonstrated that the addition of RA can induce the expression of germ cell-specific markers, in particular VASA, in a dose-dependent manner. Examination of the germ cell marker VASA, SCP3, GDF9 and TEKT1 in these experiment indicated that expression of these marker all increased in the presence and absence of RA as EB differentiation progressed. But the expression of these markers was more in the presence of RA than in the absence of RA over 5 days differentiation. 

Hence, we conclude that the use of RA may be beneficial in experiments designed to differentiate germ cells from hESCs in vitro. In addition, we also found that RA induce the expression of both GDF9 (an oocyte specific gene) and TEKT1 (a spermatid specific gene) with differentiation of the hESC line (46, XX). This suggests that RA in this culture conditions did not affect gamete determination. It maybe is the cause of the concentration of RA and the timing of adding it to the medium.
